# Status of the HIV epidemic in Manicaland, east Zimbabwe prior to the outbreak of the COVID-19 pandemic

**DOI:** 10.1371/journal.pone.0273776

**Published:** 2022-09-23

**Authors:** Adya Rao, Louisa Moorhouse, Rufu Maswera, Tawanda Dadirai, Phyllis Mandizvidza, Constance Nyamukapa, Shevanthi Nayagam, Simon Gregson

**Affiliations:** 1 Faculty of Medicine, Imperial College School of Medicine, Imperial College London, London, United Kingdom; 2 Faculty of Medicine, MRC Centre for Global Infectious Disease Analysis, School of Public Health, Imperial College London, London, United Kingdom; 3 Manicaland Centre for Public Health Research, Biomedical Research and Training Institute, Harare, Zimbabwe; 4 Department of Metabolism, Section of Hepatology & Gastroenterology, Digestion & Reproduction, Imperial College London, London, United Kingdom; Ohio University, UNITED STATES

## Abstract

**Background:**

Manicaland province in eastern Zimbabwe has a high incidence of HIV. Completion of the seventh round of the Manicaland Survey in 2018–2019 provided the opportunity to assess the state of the epidemic prior to the start of the COVID-19 pandemic. The study aims were to: a) estimate HIV seroprevalence and assess whether prevalence has declined since the last round of the survey (2012–2013), b) describe and analyse the socio-demographic and behavioural risk factors for HIV infection and c) describe the HIV treatment cascade.

**Methods:**

Participants were administered individual questionnaires collecting data on socio-demographic characteristics, sexual relationships, HIV prevention methods and treatment access, and were tested for HIV. Descriptive analyses were followed by univariate and multivariate analyses of risk factors for HIV seropositvity using logistic regression modelling based on the proximate-determinants framework.

**Results:**

HIV prevalence was 11.3% [95% CI; 10.6–12.0] and was higher in females than males up to 45–49 years. Since 2012–2013 HIV prevalence has significantly declined in 30–44 year-olds in males, and 20–44 year-olds in females. The HIV epidemic has aged since 2012–2013, with an increase in the mean age of HIV positive persons from 38 to 41 years. Socio-demographic determinants of HIV prevalence were church denomination in males, site-type, wealth-status, employment sector and alcohol use in females, and age and marital status in both sexes. Behavioural determinants associated with increased odds of HIV were a higher number of regular sexual partners (lifetime), non-regular sexual partners (lifetime) and condom use in both sexes, and early sexual debut and concomitant STIs in females; medical circumcision was protective in males. HIV status awareness among participants testing positive in our study was low at 66.2%. ART coverage amongst all participants testing positive for HIV in our study was 65.0% and was lower in urban areas than rural areas, particularly in males.

**Conclusions:**

Prevalence has declined, and ART coverage increased, since 2012–2013. Majority of the associations with prevalence hypothesised by the theoretical framework were not observed in our data, likely due to underreporting of sexual risk behaviours or the treatment-as-prevention effect of ART curtailing the probability of transmission despite high levels of sexual risk behaviour. Further reductions in HIV incidence require strengthened primary prevention, HIV testing and linkage to risk behaviour counselling services. Our results serve as a valuable baseline against which to measure the impact of the COVID-19 pandemic on HIV prevalence and its determinants in Manicaland, Zimbabwe, and target interventions appropriately.

## Introduction

Despite significant progress, HIV/AIDS continues to be one of the leading causes of death and disability globally [1,2]. The United Nations General Assembly’s “Political Declaration on HIV and AIDS” in 2016 committed to reduce global HIV infections and AIDS-related mortality to less than 500,000 by 2020 [3] and reinforced the United Nations Programme on HIV/AIDS (UNAIDS) Fast Track 90–90–90 targets for the treatment cascade [4], calling for 90% of all people living with HIV (PLHIV) to know their HIV status, 90% of those diagnosed with HIV to be on antiretroviral therapy (ART) and 90% of those receiving ART to have a suppressed viral load (<1000 copies per mL). However, all global targets remain unmet, resulting in 3.5 million additional HIV infections and 820,000 more AIDS-related deaths compared to if these targets had been achieved [5].

Although Eastern and Southern Africa has experienced a substantial reduction of 38% in HIV incidence and 49% in AIDS-related mortality from 2010 to 2019– higher than any other UNAIDS region–these declines have been slower than anticipated. This region is the most adversely impacted by HIV, contributing to over half (54%) of global PLHIV (20.6 million) in 2019 [6]. Women are disproportionately affected, with 3 in 5 new infections in 2019 occurring among women. Adolescent women (aged 15–24 years) are particularly vulnerable, being 2.5 times more likely than males of equivalent age to acquire HIV [5].

Within Eastern and Southern Africa, Zimbabwe has one of the highest burdens of HIV, with adult (≥15 years) prevalence estimated to be 13.4% [95% CI 11.8–15.3] overall, 11.3% [95% CI; 9.8–13.0] in males and 15.4% [95% CI; 13.6–17.5] in females [7]. Zimbabwe faces a generalised epidemic, sustained primarily by unprotected heterosexual sex [8]. Zimbabwe was one of the first countries in the region with a large-scale generalised epidemic to experience a decline in infection. The initial decrease in prevalence in the early 2000s was attributed to a decrease in sexual risk behaviour [9]. Between 2004 and 2015, as ART coverage increased to 72% of eligible adults, HIV prevalence continued to decline from 19.2% to 14.7% [7]. In 2016 Zimbabwe adopted the WHO “test-and-treat” strategy entailing expansion of treatment eligibility to all PLHIV regardless of CD4 count and WHO clinical stage [10,11].

Zimbabwe’s HIV/AIDS Strategic plan (2015–2020) identified Manicaland province in eastern Zimbabwe as a priority area due to its high HIV incidence [12]. Manicaland is the second most populous province after Harare, resulting in a relatively high number of PLHIV (135,137 including adults and children in 2018) [13]. It has an above average number of people living below the national poverty line and one of the lowest values of the human development index and life expectancy in the country [14].

In 1998 the Manicaland Centre for Public Health Research launched the only general population open-cohort HIV serosurvey in Zimbabwe [15]. This ongoing survey was designed to provide robust data characterising the evolving epidemiology of the HIV epidemic, assessing its impact and evaluating the effectiveness of HIV prevention and treatment programmes. Behavioural determinants of HIV prevalence in Manicaland in the past have been the number of lifetime sexual partners and non-regular partners (associated with higher odds of infection) and consistent condom use which was protective [16,17]. Socio-demographic determinants have included marital status–with higher odds in widow(er)s–and church membership (with Christian membership being protective) [18,19]. The role of socioeconomic status remains contentious, with data from 2011 finding no association between wealth and HIV infection [20], contradicting prior findings from 1998–2000 and 2001–2003 that had suggested that higher wealth was protective [21]. Round 7 –the most recent round–of the Manicaland survey was completed in 2018–19. The inter-survey period of 6 years since the previous round (2012–2013) provides scope for analysing the evolution of prevalence and its determinants to target future interventions appropriately. The impact of the scale-up of ART on the ageing of PLHIV in Zimbabwe is expected to continue [22]. The inclusion of older adults (aged 55+) in the survey for the first time provides the opportunity to assess this.

It is anticipated that the COVID-19 pandemic will influence HIV prevalence and its determinants, as well as HIV services in Manicaland. The timely completion of Round 7 of the Manicaland Survey just prior to the onset of the COVID-19 outbreak enables us to establish valuable baseline information which can later be used to measure the effect of the COVID-19 pandemic and control programmes on levels and determinants of HIV infection in Manicaland, Zimbabwe.

This study therefore aims to use data from the seventh round of the Manicaland survey to: a) estimate HIV prevalence in Manicaland in 2019 prior to the COVID-19 pandemic and assess whether prevalence has declined since the previous round of the survey (2012–2013), b) describe and analyse the socio-demographic and behavioural risk factors for HIV infection and c) describe the HIV treatment cascade.

## Materials and methods

### Study setting

Manicaland province in eastern Zimbabwe has a population of 1,752,698 people which is largely rural. It comprises 7 administrative districts and 3 town/councils, with Mutare being the capital [23].

The 2018–2019 survey was conducted across 3 districts (Mutasa, Makoni and Nyanga) and one city (Mutare). 8 sites ranging from rural to urban were sampled. Rural sites are Eastern Highlands (tea estate), Bonda Mission (subsistence farming area) and Selbourne (forestry estate); peri-urban sites are Nyazura and Nyanga (towns) and Watsomba (roadside settlement). Urban sites are Sakubva and Hobhouse. These sites represent the 5 of the major socioeconomic strata in Manicaland [15].

### Survey design and sampling frame

This was a community-based stratified cross-sectional study. The 2018–2019 survey built on the existing sampling frame [15,24,25]. The survey was conducted in two stages: an initial household census followed by enrollment of selected household members into the individual survey. A list of all eligible households in the study sites was updated based on the most recent national census (2012) [23] and generated with the aid of village community guides. All household members aged 15–24 for females and 15–29 for males, and 2/3 of randomly selected members older than this were eligible for the subsequent individual questionnaire.

### Ethical approval

Written informed consent was obtained from all participants. For participants aged under 18, written informed consent was obtained from a parent or guardian and assent was obtained from the child. The study was approved by the Imperial College Research Ethics Committee and the Medical Research Council of Zimbabwe.

### Survey implementation

The individual questionnaires collected information on socio-demographic characteristics, sexual relationships, HIV prevention methods and treatment access [26]. A validated Informal Confidential Voting Interview method was implemented, with secret voting for questions pertaining to sexual risk behaviour in order to reduce social desirability bias [27].

### HIV testing

All respondents of the individual questionnaire were asked to provide a dried blood sample (DBS) and invited for Provider Initiated Testing and Counselling (PITC) where a HIV test was conducted. For this analysis, the HIV status of the participants was defined by either i) PITC result, or ii) by the DBS test result, in patients who refused PITC and consented to a DBS. The serial HIV testing algorithm by the Ministry of Health and Childcare in Zimbabwe [28]–based on WHO recommendations [29]–was employed.

### Data analysis

Descriptive data were expressed as counts, percentages, medians and interquartile ranges. To formally compare the distribution of socio-demographic and behavioural characteristics between HIV positive and HIV negative participants, Pearson’s Chi-squared test (categorical variables) and the Mann-Whitney U test (continuous non-parametric variables) were used.

HIV prevalence was estimated by dividing the number of participants who tested positive for HIV by the number of survey participants. Estimates were weighted to account for sampling probability, stratification and clustering. 95% binomial confidence intervals were calculated. Adult prevalence was defined as prevalence in persons aged 15 and above. HIV prevalence was disaggregated by socio-demographic characteristics including age, sex, wealth status and religious affiliation. Individual wealth status was a continuous composite measure grouped into quintiles ranging from poorest to least poor using a method developed by Schur et al [20]. The categorisation of religious affiliation built upon Manzou’s 4-level categorisation of Manicaland churches [19]. HIV prevalence in 2018–2019 was also compared to that in 2012–2013 in overlapping sites.

**Fig 1:**
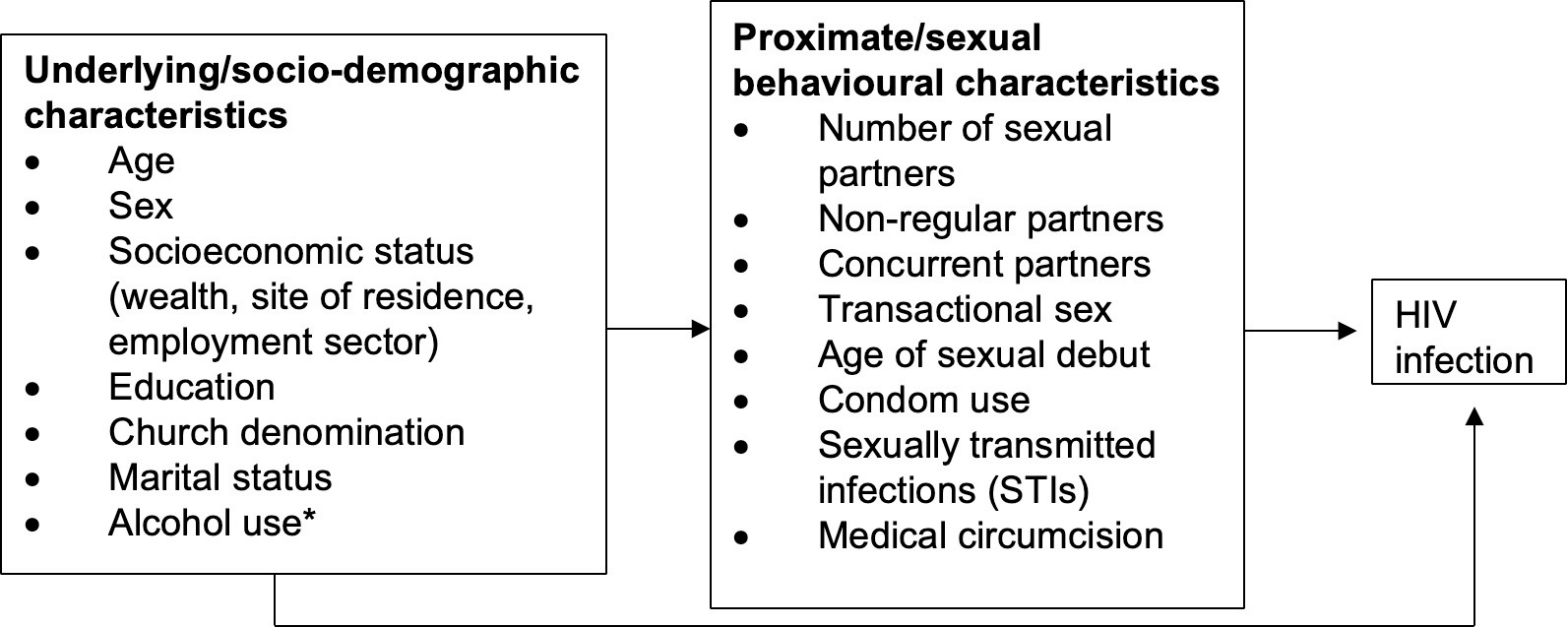
Proximate and underlying determinants of HIV infection. Socio-demographic characteristics influence HIV infection status via proximate factors (sexual risk behaviours) which in turn modify the biological risk of HIV infection. For instance, the number of sexual partners, non-regular partners, concurrent partners, transactional sex and age of sexual debut influence the probability of exposure of susceptible to infected individuals, while condom use, concomitant STIs and medical circumcision affect the efficiency of transmission per sexual contact. * Alcohol use is considered as a background factor as it is believed that its impact on HIV infection is mediated by sexual risk behaviours.

The Proximate Determinants Framework proposed by Boerma and Weir in 2005 (S1 Fig) [30] was used to construct a simplified theoretical framework (Fig 1) for the analysis of socio-demographic and behavioural associations with prevalence. Univariate analysis was conducted to identify associations of individual socio-demographic and behavioural factors with HIV infection. Analysis of sexual risk behaviours was restricted to participants reporting sexual debut. Univariate associations were then adjusted for age. Subsequently three multivariate logistic regression models were developed: (i)to test for independent associations of socio-demographic factors with HIV infection, (ii)to test for independent associations of behavioural factors with HIV infection, and (iii)to investigate whether the associations with socio-demographic factors reduce when adjusting for behavioural factors. A cut-off of p<0.1 was used to select variables after age-adjustment for inclusion in the 3 multivariate models. Weighted logistic regression analysis was conducted. Observations with data missing for a particular variable were excluded from analyses involving that variable in both descriptive analyses and regression models. P<0.05 was considered statistically significant.

For the treatment cascade, HIV status awareness and ART use were self-reported as part of the individual questionnaire. ART coverage was measured among all participants who tested positive in our study (either on PITC or DBS) and specifically among those aware of their HIV positive status. For calculation of the 90:90:90 targets, self-reported adherence was used as a proxy for viral load suppression (VLS).

All data analyses were conducted using STATA version 16.1 (STATA Corporation, USA).

## Results

### Study population

The overall survey participation rate was 73.8% (S2 Fig). Among the 9339 participants tested for HIV, 58.4% were female and 41.6% were male, with a median age of 29 years (IQR = 23). 935 participants tested positive for HIV (S1 Table).

### HIV prevalence

**Fig 2:**
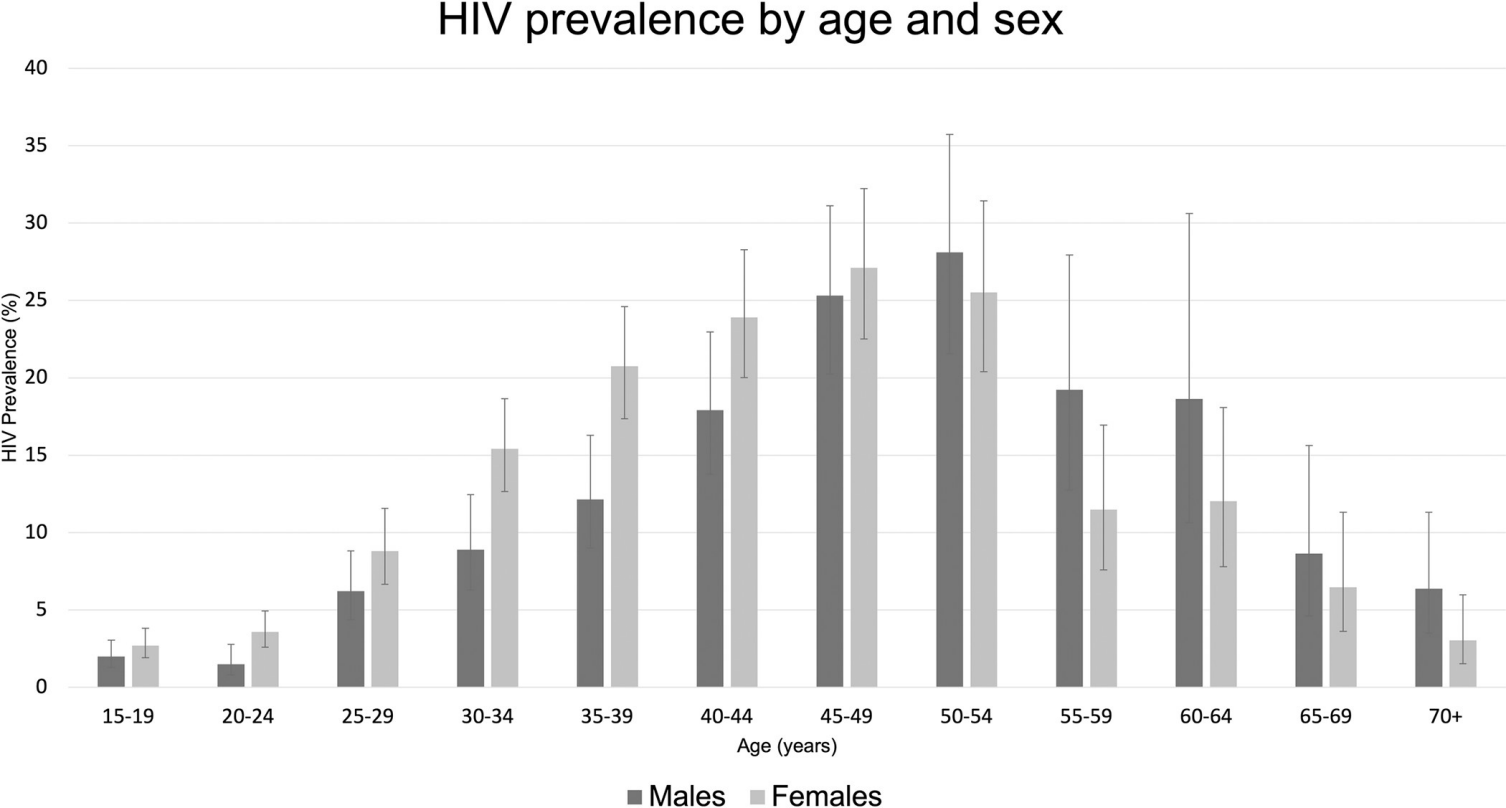
Breakdown of weighted HIV seroprevalence across socio-demographic characteristics. Error bars represent 95% CI.

The overall weighted prevalence of HIV was 11.3% [95% CI; 10.6–12.0]. Prevalence was higher in females (12.3% [95% CI; 11.4–13.2]) than in males (9.81% [95% CI; 8.85–10.86]) and this was observed in 15–49 year-olds, beyond which prevalence was higher in males (Fig 2). Females had a higher prevalence across most socio-demographic characteristics (S3 Fig).

### Comparison of prevalence between 2012–2013 and 2018–2019

**Fig 3:**
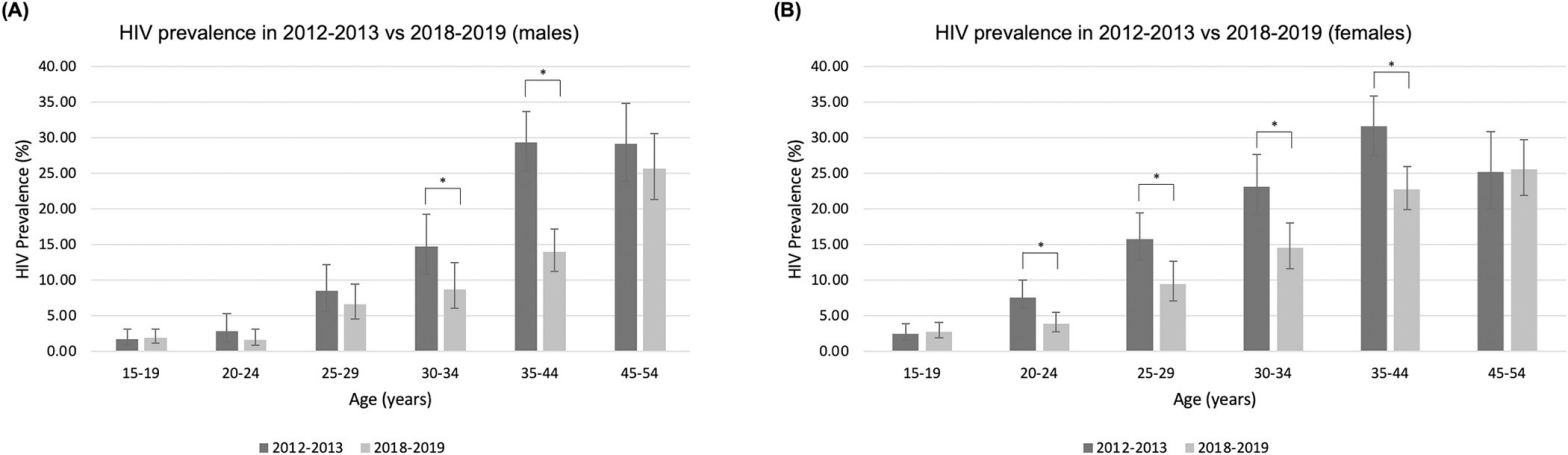
Comparison of HIV prevalence between 2012–2013 and 2018–2019 in overlapping age-groups and study sites. Error bars represent 95% CI. Asterisks (*) indicate differences that are statistically significant at the level of p<0.05.

HIV seroprevalence significantly declined between 2012–2013 and 2018–2019 in 30–34 and 35–44 year-olds in males, and 20–44 year-olds in females (Fig 3).

Between 2012–2013 and 2018–2019 the mean age of PLHIV increased from 38 to 41 years and the proportion of PLHIV aged 45–54 years increased from 24.6% to 27.1%.

### Risk factors for HIV seropositivity

Age-adjusted findings are shown in Tables 1 and 2. Model 1 (D) adjusts for demographic factors. The individual demographic factors associated with HIV infection in males were: a higher odds with increasing age (relative to 15-24s) and in currently married and widowed groups (relative to singles), and a lower odds in the Christian group. In females, site-type (higher odds in small towns relative to subsistence farming areas), wealth-status (lower odds in 4^th^ poorest compared to the poorest group), employment sector (lower odds in students compared to unemployed) and alcohol use were additionally associated, while church denomination was not. Model 2 (B) adjusts each behavioural factor for the others. The individual behavioural factors associated with HIV positivity in males were: a higher odds with increasing number of regular sexual partners (lifetime), non-regular partners and condom use, and lower odds with medical circumcision. In females, early sexual debut, a higher number of regular sexual partners (lifetime), condom use and concomitant STIs were associated with higher odds of infection.

**Table 1:** Age-adjusted and multivariate models of socio-demographic and behavioural risk factors associated with HIV prevalence in males (N = 3886).

*Factor*	*Coding*	*N*	*HIV+, n (weighted %)*	*Age-adjusted*		*Model 1 (D)*		*Model 2 (B)*		*Model 3 (D+B)*	
				aOR (95% CI)	p-value	aOR (95% CI)	p-value	aOR (95% CI)	p-value	aOR (95% CI)	p-value
***Age***	15–24	1672	30 (1.79)			1		1		1	
	25–34	803	59 (7.62)			3.98 [2.41, 6.6]	<0.001*	10.39 [3.93, 27.44]	<0.001*	8.14 [3.01, 22.03]	<0.001*
	35–44	589	87 (14.77)			8.36 [4.76, 14.67]	<0.001*	25.06 [9.35, 67.12]	<0.001*	17.22 [6.12, 48.51]	<0.001*
	45–54	398	105 (26.38)			18.88 [10.90, 32.69]	<0.001*	45.58 [17.03, 121.98]	<0.001*	34.11 [12.06, 96.44]	<0.001*
	55–64	163	31 (19.02)			10.53 [5.46, 20.30]	<0.001*	26.29 [9.17, 75.32]	<0.001*	15.16 [4.85, 47.4]	<0.001*
	65+	261	19 (7.28)			3.03 [1.42, 6.47]	0.004*	#		#	
***Site type***	Subsistence farming	616	47 (8.68)	1							
	Small towns	864	74 (9.85)	1.16 [0.77, 1.74]	0.471						
	Estates	1222	119 (11.15)	1.21 [0.84, 1.77]	0.296						
	Roadside settlements	633	46 (8.22)	1.00 [0.64, 1.56]	0.996						
	Urban	551	45 (9.89)	1.34 [0.85, 2.11]	0.208						
***Wealth status***	Poorest	379	26 (7.98)	1		1				1	
	2^nd^ poorest	1872	175 (10.62)	1.48 [0.95, 2.31]	0.082	1.47 [0.94, 2.30]	0.090			1.28 [0.77, 2.13]	0.345
	3^rd^ poorest	855	77 (10.46)	1.37 [0.84, 2.22]	0.204	1.36 [0.84, 2.21]	0.210			1.36 [0.78, 2.36]	0.279
	4^th^ poorest	733	51 (8.20)	1.09 [0.66, 1.82]	0.737	1.13 [0.68, 1.90]	0.638			0.93 [0.52, 1.65]	0.794
	Least poor	47	2 (5.41)	0.70 [0.16, 3.09]	0.640	0.75 [0.17, 3.44]	0.716			0.90 [0.19, 4.33]	0.899
***Highest education level***	None/ primary	618	74 (15.88)	1							
	Secondary/ higher	3268	257 (10.29)	0.77 [0.56, 1.07]	0.123						
***Employment sector***	Formal	1091	115 (11.79)	1							
	Informal	896	99 (12.10)	1.11 [0.83, 1.50]	0.475						
	Student	827	15 (1.81)	1.06 [0.54, 2.10]	0.868						
	Unemployed	1072	102 (10.67)	1.22 [0.90, 1.65]	0.190						
***Marital status***	Never married	1652	45 (2.98)	1		1				1	
	Currently married	2033	239 (12.53)	1.01 (0.64, 1.61]	0.963	0.98 [0.62, 1.55]	0.943			2.57 [1.30, 5.05]	0.006*
	Divorced/ separated	137	28 (20.95)	2.00 [1.07, 3.72]	0.030*	1.88 [1.00, 3.51]	0.048*			1.46 [0.61, 3.46]	0.391
	Widowed	64	19 (29.84)	4.68 [2.13, 10.24]	<0.001*	4.49 [2.03, 9.95]	<0.001*			20.96 [5.79, 75.8]	<0.001*
***Church denomination***	Christian	1927	139 (8.23)	1		1				1	
	Spiritualist (including Apostolic)	984	80 (9.60)	1.16 [0.86, 1.56]	0.339	1.09 [0.80, 1.48]	0.571			1.40 [0.97, 2.02]	0.069
	Other	441	40 (10.63)	1.30 [0.88, 1.92]	0.183	1.26 [0.85, 1.87]	0.248			1.56 [0.97, 2.49]	0.064
	None	534	72 (14.96)	1.71 [1.24, 2.36]	0.001*	1.63 [1.17, 2.26]	0.004*			1.76 [1.18, 2.63]	0.006*
***Frequent alcohol use***	No	2538	193 (8.91)	1							
	Yes	1348	138 (11.38)	0.96 [0.76, 1.23]	0.767						
***Age of sexual debut***	18+	2152	245 (12.23)	1				1		1	
	≤17	573	60 (12.38)	1.45 [1.05, 2.01]	0.024*			1.01 [0.69, 1.50]	0.06	1.02 [0.68, 1.52]	0.938
***Number of regular sexual partners (lifetime)***	1	1319	103 (8.38)	1				1		1	
	2–4	864	137 (17.05)	1.95 [1.47, 2.58]	<0.001*			1.54 [1.13, 2.10]	0.006*	1.44 [1.04, 1.98]	0.027*
	5+	238	49 (22.40)	3.14 [2.13, 4.64]	<0.001*			2.15 [1.38, 3.36]	0.001*	2.26 [1.45, 3.50]	<0.001*
***Non-regular sexual partners (lifetime)***	No	968	78 (8.70)	1				1		1	
	Yes	1757	227 (14.26)	1.3 [1.38, 2.43]	<0.001*			1.44 [1.03, 2.01]	0.031*	1.42 [1.01, 2.01]	0.044*
***Multiple sexual partners (last year)***	No	4329	544 (12.08)	1				1		1	
	Yes	134	36 (13.35)	1.41 [0.99, 2.01]	0.060			0.93 [0.62, 1.41]	0.737	1.01 [0.66, 1.54]	0.050
***Multiple sexual partners (last month)***	No	966	97 (11.19)	1							
	Yes	1759	208 (12.82)	0.88 [0.67, 1.15]	0.346						
***Concurrent sex***	No	2555	283 (12.12)	1							
	Yes	170	22 (14.41)	1.18 [0.72, 1.97]	0.497						
***Transactional sex***	No	2017	205 (11.34)	1				1		1	
	Yes	460	83 (19.43)	1.72 [1.29, 2.30]	<0.001*			1.18 [0.84, 1.64]	0.336	1.13 [0.80, 1.61]	0.495
***Condom use throughout last sexual encounter***	No	2014	159 (8.40)	1				1		1	
	Yes	711	146 (24.76)	6.92 [5.17, 9.26]	<0.001*			5.58 [4.10, 7.60]	<0.001*	7.28 [5.03, 10.53]	<0.001*
***Medical circumcision***	No	3069	321 (11.69)	1				1		1	
	Yes	816	10 (1.53)	0.23 [0.12, 0.44]	<0.001*			0.34 [0.16, 0.69]	0.003*	0.36 [0.17, 0.74]	0.006*
***STI symptoms***	No	3767	316 (8.68)	1							
	Yes	119	15 (9.10)	1.48 [0.82, 2.65]	0.192						

Univariate, age-adjusted and multivariate models are shown in succession. Model 1 (D) includes all socio-demographic characteristics and model 2 (B) includes all sexual risk behaviours that met the cut-off for significance (p<0.1) in the age-adjusted model. Model 3 (D+B) includes all these behavioural characteristics and the socio-demographic characteristics that maintained model fit. Multiple sexual partners in the last month was unable to be added to Model 2 as it was expected a priori to be collinear with multiple partners last year. # Observations were dropped from these categories due to low numbers of participants testing positive for HIV.

**Table 2:** Age-adjusted and multivariate models of socio-demographic and behavioural risk factors associated with HIV prevalence in females (N = 5453).

*Factor*	*Coding*	*N*	*HIV+, n (weighted %)*	*Age-adjusted*		*Model 1 (D)*		*Model 2 (B)*		*Model 3 (D+B)*	
				aOR (95% CI)	p-value	aOR (95% CI)	p-value	aOR (95% CI)	p-value	aOR (95% CI)	p-value
***Age***	15–24	2148	67 (3.12)			1		1		1	
	25–34	1080	132 (12.22)			4.41 [3.03, 6.41]	<0.001*	3.36 [2.28, 4.94]	<0.001*	3.36 [2.21, 5.11]	<0.001*
	35–44	892	198 (22.20)			9.15 [6.32, 13.3]	<0.001*	6.56 [4.49, 9.57]	<0.001*	6.58 [4.34, 9.97]	<0.001*
	45–54	560	148 (26.43)			8.29 [5.54, 12.39]	<0.001*	8.86 [5.90, 13.3]	<0.001*	6.23 [3.98, 9.76]	<0.001*
	55–64	341	40 (11.73)			2.22 [1.28, 2.86]	0.004*	4.10 [2.50, 6.70]	<0.001*	2.12 [1.17, 3.85]	0.014*
	65+	432	19 (4.40)			0.50 [0.25, 1.00]	0.05	1.59 [0.20, 12.62]	0.659	0.38 [0.05, 3.10]	0.364
***Site type***	Subsistence farming	858	81 (8.68)	1		1				1	
	Small towns	1470	179 (9.85)	1.40 [1.04, 1.87]	0.023*	1.71 [1.24, 2.37]	0.001*			1.57 [1.09, 2.26]	0.016*
	Estates	1140	143 (11.15)	1.36 [1.01, 1.85]	0.041*	1.59 [1.13, 2.24]	0.008*			1.27 [0.87, 1.87]	0.216
	Roadside settlements	1023	108 (8.22)	1.20 [0.88, 1.64]	0.253	1.30 [0.93, 1.80]	0.121			1.30 [0.90, 1.88]	0.157
	Urban	962	93 (9.89)	1.26 [0.91, 1.75]	0.166	1.77 [1.20, 2.62]	0.004*			1.50 [0.96, 2.35]	0.071
***Wealth status***	Poorest	544	68 (13.43)	1		1				1	
	2^nd^ poorest	2347	289 (13.55)	0.96 [0.71, 1.29]	0.767	0.88 [0.64, 1.21]	0.429			0.90 [0.63, 1.30]	0.591
	3^rd^ poorest	1247	133 (11.99)	0.82 [0.59, 1.13]	0.226	0.71 [0.49, 1.03]	0.068			0.75 [0.49, 1.14]	0.181
	4^th^ poorest	1231	108 (9.83)	0.65 [0.46, 0.92]	0.014*	0.56 [0.37, 0.84]	0.005*			0.65 [0.40, 1.03]	0.069
	Least poor	81	6 (8.53)	0.54 [0.22, 1.33]	0.183	0.49 [0.19, 1.24]	0.132			0.80 [0.27, 2.35]	0.682
***Highest education level***	None/ primary	1472	198 (14.05)	1		1				1	
	Secondary/ higher	3981	406 (11.54)	0.75 [0.60, 0.94]	0.011*	0.92 [0.72, 1.17]	0.485			0.95 [0.73, 1.25]	0.732
***Employment sector***	Unemployed	3040	357	1		1				1	
	Formal	513	84	0.93 [0.71, 1.21]	0.586	0.82 [0.61, 1.10]	0.180			0.72 [0.51, 1.02]	0.067
	Informal	1107	143	0.86 [0.69, 1.07]	0.185	0.81 [0.65, 1.02]	0.069			0.77 [0.60, 1.00]	0.051
	Student	793	20	0.63 [0.37, 1.06]	0.080	0.54 [0.30, 0.97]	0.040*			0.69 [0.22, 2.27]	0.546
***Marital status***	Never married	1297	66 (6.16)	1		1				1	
	Currently married	3100	295 (10.24)	0.61 [0.43, 0.87]	0.007*	0.53 [0.37, 0.77]	0.001*			0.80 [0.49, 1.30]	0.362
	Divorced/ separated	479	103 (22.47)	1.58 [1.05, 2.39]	0.028*	1.29 [0.85, 1.95]	0.234			0.92 [0.55, 1.56]	0.771
	Widowed	577	140 (24.33)	3.08 [1.97, 4.82]	<0.001*	2.60 [1.67, 4.05]	<0.001*			4.13 [2.32, 7.37]	<0.001*
***Church denomination***	Christian	3035	314 (11.44)	1		1				1	
	Spiritualist (including Apostolic)	1594	182 (12.87)	1.07 [0.87, 1.31]	0.522	0.97 [0.78, 1.21]	0.799			0.89 [0.70, 1.14]	0.353
	Other	668	82 (13.81)	1.05 [0.80, 1.39]	0.705	0.99 [0.74, 1.33]	0.972			0.84 [0.60, 1.18]	0.306
	None	156	26 (18.48)	2.02 [1.25, 3.28]	0.004*	1.50 [0.91, 2.46]	0.112			1.07 [0.59, 1.91]	0.833
***Frequent alcohol use***	No	5302	573 (12.00)	1		1				1	
	Yes	149	31 (22.14)	2.40 [1.52, 3.79]	<0.001*	1.97 [1.25, 3.12]	0.004*			1.20 [0.68, 2.15]	0.528
***Age of sexual debut***	18+	3022	369 (12.85)	1				1		1	
	<18	1444	212 (16.27)	1.68 [1.39, 2.04]	<0.001*			1.43 [1.15, 1.77]	0.001*	1.39 [1.10, 1.76]	0.005*
***Number of regular sexual partners (lifetime)***	1	3156	232 (7.85)	1				1		1	
	2–4	1093	316 (30.03)	4.26 [3.52, 5.17]	<0.001*			3.11 [2.51, 3.85]	<0.001*	3.07 [2.45, 3.85]	<0.001*
	5+	81	24 (31.42)	4.60 [2.70, 7.83]	<0.001*			2.26 [1.18, 4.34]	0.015*	2.26 [1.11, 4.61]	0.024*
***Non-regular sexual partners (lifetime)***	No	3422	391 (12.16)					1		1	
	Yes	1043	190 (19.80)	1.80 [1.48, 2.21]	<0.001*			1.16 [0.91, 1.48]	0.228	1.09 [0.84, 1.41]	0.537
***Multiple sexual partners (last year)***	No	4329	544 (13.45)					1		1	
	Yes	134	36 (29.24)	3.61 [2.31, 5.68]	<0.001*			1.11 [0.60, 2.07]	0.730	0.97 [0.50, 1.88]	0.927
***Multiple sexual partners (last month)***	No	2143	318 (15.76)								
	Yes	2322	263 (12.15)	0.65 [0.54, 0.78]	<0.001*						
***Concurrent sex***	No	4415	565 (13.70)					1		1	
	Yes	50	16 (33.08)	3.46 [1.71, 7.02]	0.001*			1.30 [0.52, 2.38]	0.575	1.35 [0.51, 3.61]	0.549
***Transactional sex***	No	3700	468 (13.66)					1		1	
	Yes	365	95 (27.96)	2.55 [1.95, 3.34]	<0.001*			0.91 [0.65, 1.29]	0.607	0.80 [0.55, 1.17]	0.248
***Condom use throughout last sexual encounter***	No	3795	330 (9.28)					1		1	
	Yes	670	251 (39.99)	5.96 [4.85, 7.32]	<0.001*			4.34 [3.43, 5.47]	<0.001*	4.70 [3.62, 6.10]	<0.001*
***STI symptoms***	No	5077	529 (10.66)	1				1		1	
	Yes	376	75 (18.02)	2.02 [1.52, 2.70]	<0.001*			1.67 [1.20, 2.32]	0.002*	1.87 [1.33, 2.64]	<0.001*

Univariate, age-adjusted and multivariate models are shown in succession. Model 1 (D) includes all socio-demographic characteristics and model 2 (B) includes all sexual risk behaviours that met the cut-off for significance (p<0.1) in the age-adjusted model. Model 3 (D+B) includes all these behavioural characteristics and the socio-demographic characteristics that maintained model fit. Multiple sexual partners in the last month was unable to be added to Model 2 as it was expected a priori to be collinear with multiple partners last year.

Model 3 (D+B) adjusts for socio-demographic and behavioural covariates to assess whether behavioural variables explain the associations of HIV infection with socio-demographic variables in line with the theoretical framework. With respect to background/socio-demographic variables in males, the highest odds of infection was in those aged 45–54 (compared to 15-24s) (aOR = 34.11; 95% CI 12.06–96.44, p<0.001), those who were widowed (relative to single) (aOR = 20.96; 95% CI 5.79–75.8, p<0.001) and don’t belong to a church (aOR = 1.76; 95% CI 1.18–2.63, p = 0.006). In females, the highest odds of HIV was in those aged 35–44 (relative to 15-24s) (aOR = 6.58; 95% CI 4.34–9.97, p<0.001), living in small towns (relative to subsistence farming areas) (aOR = 1.57; 95% CI 1.09–2.26, p = 0.016), and those who were widowed (relative to singles) (aOR = 4.13; 95% CI 2.32–7.37, p<0.001). The proximate/behavioural factors in males associated with the highest odds of infection were having 5+ regular sexual partners (relative to 1 sexual partner) (aOR = 2.26; 95% CI 1.45–3.50, p<0.001), any non-regular partners in a lifetime (aOR = 1.42; 95% 1.01–2.01, p = 0.044) and condom use (aOR = 7.28; 95% 5.03–10.53, p<0.001); medical circumcision was protective (aOR = 0.36; 95% CI 0.17–0.74, p = 0.006). In females, early sexual debut (aOR = 1.39; 95% CI 1.10–1.76, p = 0.005), 2–4 regular sexual partners (relative to 1 sexual partner) (aOR = 3.07; 95% CI 2.45–3.85, p<0.001), condom use (aOR = 4.70, 95% CI 3.62–6.10) and concomitant STIs (aOR = 1.87; 95% CI 1.33–2.64) were associated with the highest odds of infection.

Results across the different models suggest that in males the higher odds in older age-groups, in currently married and widowed groups and in those with no church membership became more apparent after adjusting for behavioural covariates. Meanwhile, the higher odds in the divorced/separated group was explained by sexual risk behaviour. In females, all the associations with socio-demographic factors were explained by sexual risk behaviour except the higher odds in the widowed group which became more apparent upon adjustment for sexual risk behaviour.

### HIV treatment cascade

Among participants who tested positive for HIV in our study (N = 935), 619 (66.2%) had reported previously testing positive. Of those aware of their diagnosis, 593 (95.8%) were on ART, of which 583 (98.3%) reported taking ART every day or on most days. However, when taking into account ART coverage amongst all those who were HIV positive (whether they were aware of their diagnosis or not), this was lower at 65.0% (608/935).

**Fig 4:**
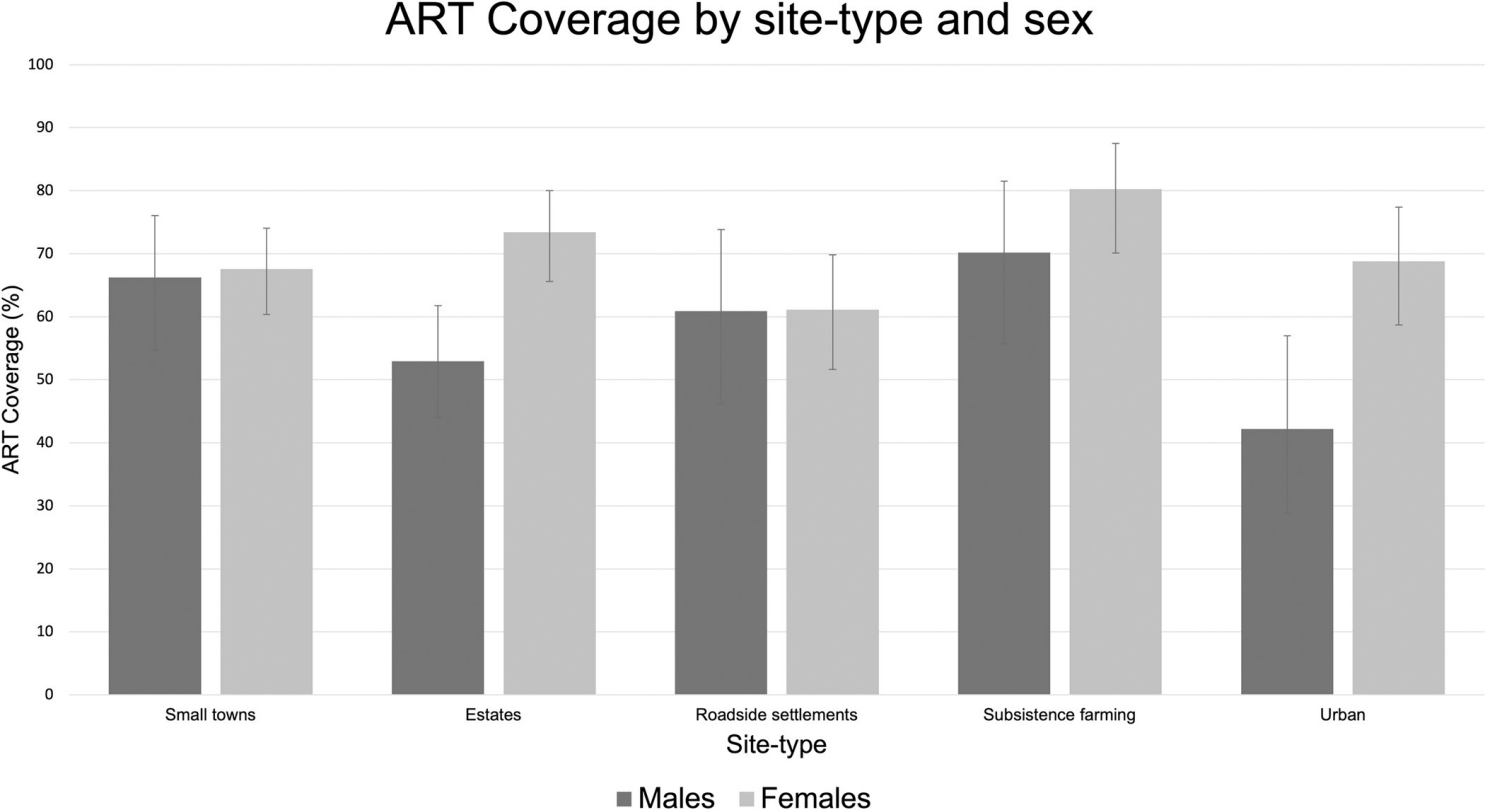
Breakdown of ART coverage by site-type and sex. ART coverage is shown among participants who tested positive in our study, regardless of HIV status awareness or previous diagnosis. Error bars represent 95% CI. Asterisks (*) indicate differences that are statistically significant at the level of p<0.05.

ART coverage among those who tested positive in our study was higher in females than males (p<0.001) and increased with increasing age in males (OR = 1.03; 95% CI [1.03–1.04], p<0.001) and females (OR = 1.02; 95% CI [1.02–1.02], p<0.001). ART coverage did not differ significantly between urban and rural areas in males (p = 0.240) and females (p = 0.878) (Fig 4).

## Discussion

Our study found that the 2018–2019 adult prevalence of HIV in Manicaland was 11.3% [95% CI; 10.6–12.0]. This represents a sustained reduction in prevalence from 15.8% in the survey 6 years prior, a trend that was seen in most age-groups and both sexes in overlapping survey sites. Our prevalence estimate for 2018–2019 was similar to that of 10.2% [95% CI: 8.6–11.9] found for adults in Manicaland province in the 2020 national ZIMPHIA survey [31]. We also found a substantial increase in ART coverage among PLHIV from 38.2% in 2012–2013 to 65.0% in 2018–2019, and that the second and third of the UNAIDS 90:90:90 targets were achieved in Manicaland (66.2: 95.8: 98.3).

These results reflect the remarkable progress that has been made towards controlling the HIV epidemic in Manicaland province over the last few decades. However, caution must be taken that this promising trajectory is not derailed as a result of the COVID-19 pandemic, both in Manicaland and the wider region. There is a myriad of mechanisms through which COVID-19 pandemic may disrupt HIV control. Firstly, it may influence sexual risk behaviours and the HIV treatment cascade. For instance, consistent with previous literature, we found young women to be disproportionately affected by HIV. Extended periods of home confinement may render young women more susceptible to sexual abuse, potentially amplifying this gender disparity [5]. Furthermore, country-wide school closures may interrupt education that is potentially protective against HIV [32]. Access to HIV services may also be disrupted due to the diversion of financial resources towards COVID-19 and the economic distress stemming from lockdowns. In Zimbabwe the number of voluntary male medical circumcision procedures fell from 24000 in February 2020 to several hundred around April 2020 [33]. The number of African countries reporting ART disruptions increased from 4 to 8 between November 2020 and March 2021 [34]. A three-month interruption for 40% of those on ART could cause a similar number of additional deaths to those that might be avoided from COVID-19 through social distancing measures in sub-Saharan Africa [35].

We found HIV prevalence to be highest in people aged 50–54 (Fig 2A), likely reflecting a greater number of cumulative lifetime sexual partners relative to younger age-groups [36]; in line with this, we found the number of regular sexual partners (lifetime) to be associated with higher odds of HIV infection in both sexes. The falling prevalence in adults aged 55+ reflects lower incidence coupled with past high mortality [37,38]. Our findings also point towards the ageing of the HIV epidemic in Manicaland, which has previously been attributed to increased survival with expansion of ART coverage and incident infections arising from increased sexual risk behaviours in older age-groups (e.g. lower condom use) [19]. The prevalence of 9.11% reported in adults aged 55+ for the first time during the Manicaland survey is relevant for future assessment of this ageing epidemic.

The female:male ratio was 1.25 and this disparity was largest among 20–24 year-olds, reinforcing previous findings that young women are particularly vulnerable to HIV [37,39]. This may stem from particular sexual risk behaviours observed in women, especially at younger ages [40,41]. We found that having non-regular partners (lifetime) and not being circumcised were risk-factors exclusive to males, and early sexual debut and STI symptoms were exclusive to females. This aligns with previous studies suggesting that in sub-Saharan Africa the higher biological susceptibility to HIV and STIs in females is compounded by behavioural, socioeconomic and structural factors including earlier sexual debut, lower condom use, age-disparate relationships, transactional sex, gender inequality and gender-based violence [39,41]. As prevalence is influenced by mortality, increasing ART coverage coupled with higher adherence in women may contribute to higher mortality in men than women, thus amplifying this gender disparity in the future, in keeping with findings from other countries in sub-Saharan Africa [39].

The associations of the aforementioned sexual risk behaviours with HIV seropositivity showed varying consistency with literature. Historically, key behavioural determinants of prevalence for this cohort have been the number of lifetime sexual partners, the presence of STI cofactors and local HIV prevalence in women (for men) [42]. We did not assess the latter. The lack of association with STI cofactors in men may stem from lower male participation rates and reporting of STI symptoms [15]. The self-reported nature of this variable and the prevalence of asymptomatic STIs contribute to further unreliability of this metric [30]. The protective effect found for male medical circumcision is in keeping with 3 randomised controlled trials in sub-Saharan Africa that found its efficacy in preventing the risk of HIV transmission to be greater than 60% [43–45].

Underlying/socio-demographic factors independently associated with HIV infection were: church denomination for males, site-type, wealth-status, employment sector and alcohol for females, and age and marital status in both sexes. The higher prevalence in widow(er)s mirrors early patterns in Manicaland which may reflect that widow(er)s’ former partners died of HIV-related illnesses [46,47]. Previous studies suggest that widow(er)s may contribute to onward transmission by re-entering high-risk sexual partnerships [18], emphasising the importance of reducing prevalence in this group. In contrast to widowhood, we did not find divorce to be a predictor of HIV prevalence, in line with recent findings in Manicaland [47].

Christian church membership was protective compared to no religion in men only. The lack of association with Spiritualist religion in both sexes contrasts prior findings [19]. It was postulated that the protective effect of Christian membership arose from the advocacy of safe-sex practices and that of Spiritualist membership from rules prohibiting extra-marital sexual relations and alcohol consumption [21]. Examining trends in church teachings and practices since 2005 may better elicit reasons for current observed findings.

Multiple associations hypothesised by our theoretical framework were not observed within our data. Several factors may explain this. Underreporting of measured sexual risk behaviours is likely and is reflected in the spurious association found between condom use and HIV infection despite adjustment for sexual behaviours. Furthermore, our model does not capture other important variables: untreated opposite-sex prevalence and ART coverage would modify the probability of exposure of susceptible to infected individuals [21]. With increased ART coverage over time, sexual risk behaviours may not be translating into the same degree of transmission as the pre-ART period, pointing towards the treatment-as-prevention effect of ART [48].

Although we found improvements in the HIV treatment cascade since 2012–2013, several gaps remain. Firstly, VLS is likely an overestimate as self-reported adherence is subject to recall and social desirability bias[49]. Furthermore, our finding of low HIV status awareness among PLHIV calls for strengthened HIV testing services. The low rate of HIV status awareness found in our study is despite an increase in the number of health facilities offering free HIV testing and counselling from 2013 to 2015 in eastern Zimbabwe, highlighting the need for further studies assessing the determinants of testing uptake [50]. It has been suggested that STI and antenatal care can be leveraged to increase testing uptake in males and females respectively. Our finding of low rates of self-testing emphasises the scope for expansion in this area [50,51].

Major strengths of our study include the large sample size (N = 9339), the sampling of 5 major socioeconomic strata in Manicaland with the addition of 2 urban sites increasing the representativeness of the sample, the Informal Confidential Voting Interview system implemented for sensitive questions to minimise social desirability bias and the low amounts of missing data due to electronic data collection preventing questions from being skipped. A key limitation was its cross-sectional nature, precluding any inferences about the causality of factors associated with HIV infection. Future studies measuring incidence are warranted and would enable comparison of risk factors for prevalence with those for incidence [34]. Additionally, we were unable to objectively quantify VLS, underestimating the risk of onward transmission. Lastly, performing multilevel regression modelling would facilitate more robust analysis of risk factors at the individual, contextual and national level [47].

Overall, we found a decrease in HIV prevalence and an increase in ART coverage since 2012–2013. However, the increase in ART coverage alone is not sufficient and must be coupled with enhanced testing and linkage to risk behaviour counselling services, as well as with strengthened primary prevention programmes in order to achieve desired reductions in incidence. Our findings serve as valuable baseline information against which to measure the impact of the COVID-19 pandemic and control programmes on levels and determinants of HIV infection in Manicaland.

## Supporting information

S1 Fig Proximate determinants framework proposed by Boerma and Weir in 2005. Underlying determinants influence the incidence of HIV via a number of proximate determinants and corresponding biological determinants. The prevalence of HIV infection feeds back into the biological determinants as it influences the probability of exposure of susceptible to infected individuals.
(DOCX)

S2 Fig Participant flowchart. Out of 12,651 participants initially eligible for Round 7 of the individual questionnaire, 9339 participants consented to have a HIV test, either via Provider Initiated Testing and Counseling (PITC) or by providing a dried blood sample (DBS). *Participants were allowed to provide multiple reasons.
(DOCX)

S3 Fig Breakdown of weighted HIV seroprevalence across socio-demographic characteristics. Error bars represent 95% CI.
(DOCX)

S1 Table Socio-demographic characteristics and risk behaviours of study population by HIV status. PrEP = Pre-exposure prophylaxis. P-values represent results of Chi-squared (categorical) or Mann-Whitney U tests (continuous, non-parametric). Asterisks (*) represents p<0.05. Where denominators are less than N, missing data is truly missing (omitted due to low numbers) and/or arising from skip rules in the questionnaire (explained below):
δ Restricted to participants currently not enrolled in school, including those aged 65+.
ε Alcohol drinkers included participants who reported having any drink in the past year or having been to a bar/beer hall in the past month.
^ϕ^ Questions on sexual partners were restricted to those who reported ever having sex.
^**ς**^ Restricted to participants reporting at least one sexual partner in the last year.
γ Transactional sex was defined as having ever been involved in a non-marital relationship where participants gave something in exchange for sex or having given/received money in exchange for sex in participants’ past 3 sexual relationships. This was restricted to participants aged <65 years.
^ψ^ Self-reported STI symptoms in the last 12 months including discharge, pain and genital sores; restricted to sexually active participants.
σ Results shown for those participants who reported having at least one non-regular sexual partner in their lifetime or of their past 3 sexual partners.
(DOCX)
