# Supplementary material for: Status of the HIV epidemic in Manicaland, east Zimbabwe prior to the outbreak of the COVID-19 pandemic
Source: PLoS One. 2022 Sep 23;17(9):e0273776. doi: 10.1371/journal.pone.0273776 (PMC9506661; doi:10.1371/journal.pone.0273776)
Supplement: S1 Fig — Underlying determinants influence the incidence of HIV via a number of proximate determinants and corresponding biological determinants. The prevalence of HIV infection feeds back into the biological determinants as it influences the probability of exposure of susceptible to infected individuals. (DOCX) [file pone.0273776.s001.docx]

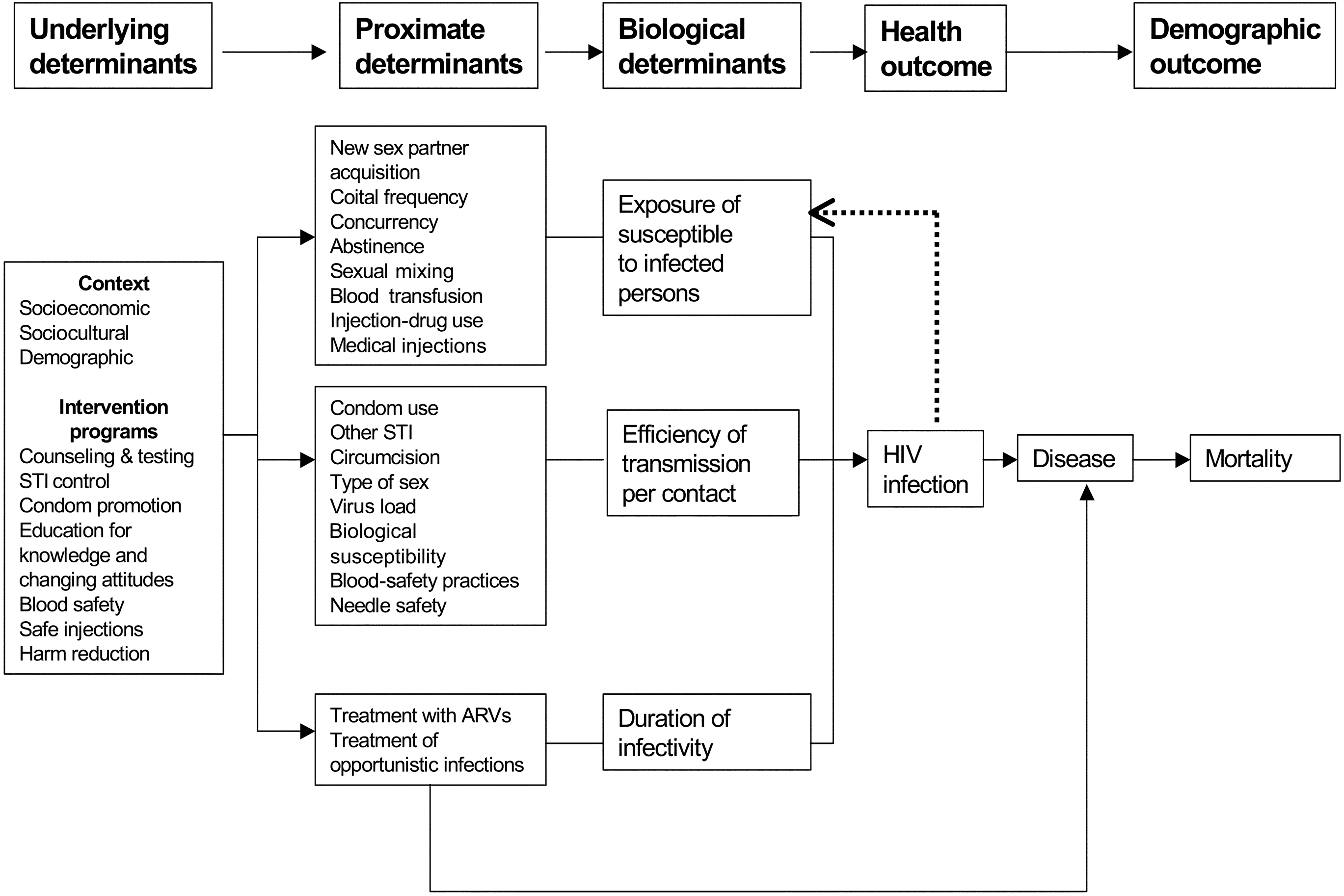


**S1 Fig. Proximate Determinants Framework proposed by Boerma and Weir in 2005.** Underlying determinants influence the incidence of HIV via a number of proximate determinants and corresponding biological determinants. The prevalence of HIV infection feeds back into the biological determinants as it influences the probability of exposure of susceptible to infected individuals.
