# Supplementary material for: Status of the HIV epidemic in Manicaland, east Zimbabwe prior to the outbreak of the COVID-19 pandemic
Source: PLoS One. 2022 Sep 23;17(9):e0273776. doi: 10.1371/journal.pone.0273776 (PMC9506661; doi:10.1371/journal.pone.0273776)
Supplement: S2 Fig — Out of 12,651 participants initially eligible for Round 7 of the individual questionnaire, 9339 participants consented to have a HIV test, either via Provider Initiated Testing and Counseling (PITC) or by providing a dried blood sample (DBS). *Participants were allowed to provide multiple reasons. (DOCX) [file pone.0273776.s002.docx]

**Participants eligible for Round 7 individual questionnaire**

(N=12,651)

**Respondents of Round 7 questionnaire**

(n=9,803)

**Did not consent** (n=367)*

1. Insufficient time (n=59)
2. DBS samples (n=4)
3. Information too personal (n=92)
4. Other (n=217)

**Did not attend individual survey** (n=2481)

**Consented for DBS and/or PITC**

(n=9,339)

**S2 Fig. Participant flowchart.** Out of 12,651 participants initially eligible for Round 7 of the individual questionnaire, 9339 participants consented to have a HIV test, either via Provider Initiated Testing and Counseling (PITC) or by providing a dried blood sample (DBS). *Participants were allowed to provide multiple reasons.
