# Supplementary material for: Status of the HIV epidemic in Manicaland, east Zimbabwe prior to the outbreak of the COVID-19 pandemic
Source: PLoS One. 2022 Sep 23;17(9):e0273776. doi: 10.1371/journal.pone.0273776 (PMC9506661; doi:10.1371/journal.pone.0273776)
Supplement: S3 Fig — Error bars represent 95% CI. (DOCX) [file pone.0273776.s003.docx]

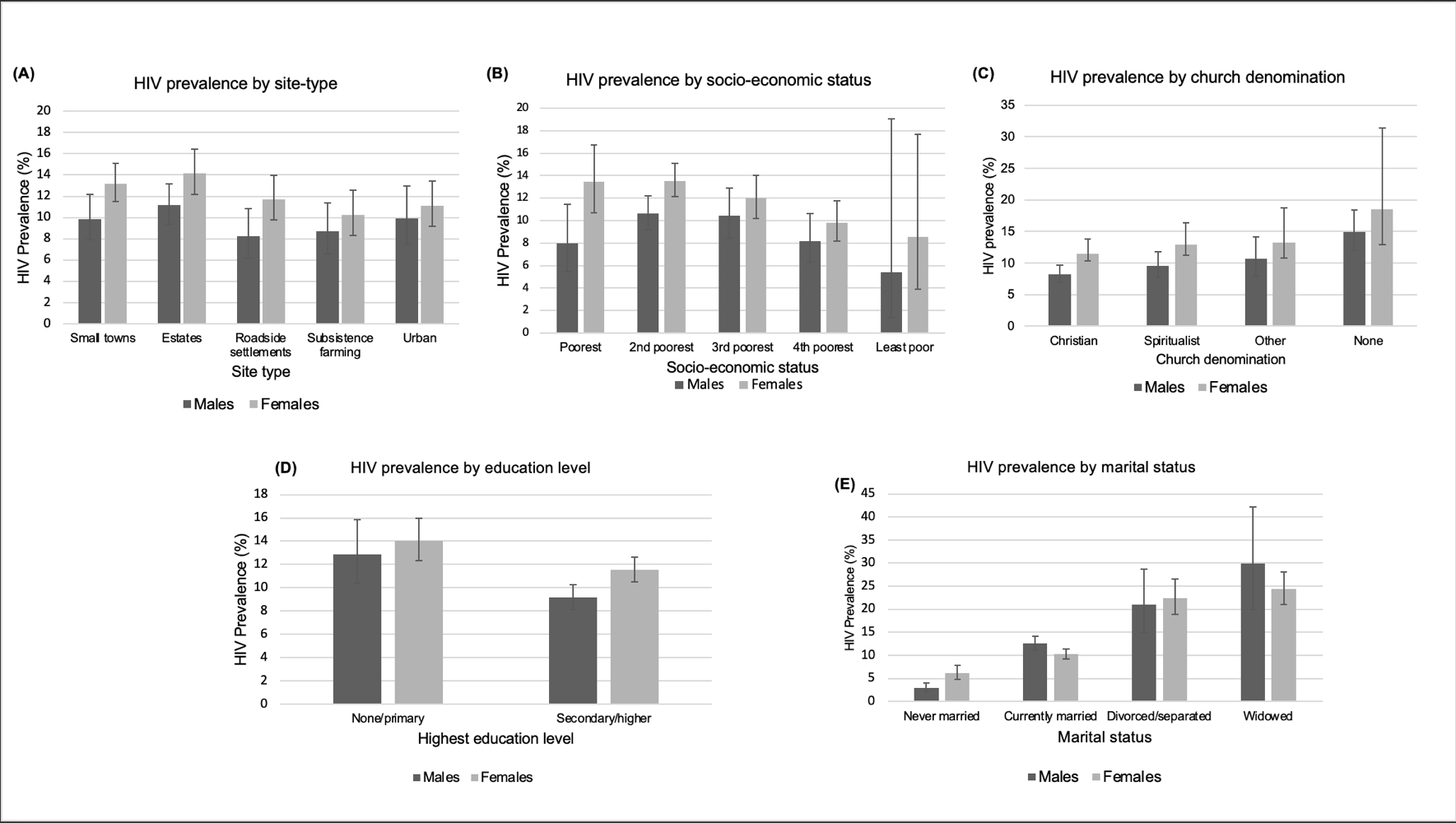


**S3 Fig. Breakdown of weighted HIV seroprevalence across socio-demographic characteristics**. Error bars represent 95% CI.
