# Supplementary material for: Status of the HIV epidemic in Manicaland, east Zimbabwe prior to the outbreak of the COVID-19 pandemic
Source: PLoS One. 2022 Sep 23;17(9):e0273776. doi: 10.1371/journal.pone.0273776 (PMC9506661; doi:10.1371/journal.pone.0273776)
Supplement: S1 Table — PrEP = Pre-exposure prophylaxis. P-values represent results of Chi-squared (categorical) or Mann-Whitney U tests (continuous, non-parametric). Asterisks (*) represents p<0.05. Where denominators are less than N, missing data is truly missing (omitted due to low numbers) and/or arising from skip rules in the questionnaire (explained below): δ Restricted to participants currently not enrolled in school, including those aged 65+. ε Alcohol drinkers included participants who reported having any drink in the past year or having been to a bar/beer hall in the past month. ϕ Questions on sexual partners were restricted to those who reported ever having sex. ς Restricted to participants reporting at least one sexual partner in the last year. γ Transactional sex was defined as having ever been involved in a non-marital relationship where participants gave something in exchange for sex or having given/received money in exchange for sex in participants’ past 3 sexual relationships. This was restricted to participants aged <65 years. ψ Self-reported STI symptoms in the last 12 months including discharge, pain and genital sores; restricted to sexually active participants. σ Results shown for those participants who reported having at least one non-regular sexual partner in their lifetime or of their past 3 sexual partners. (DOCX) [file pone.0273776.s004.docx]

| Characteristics | Total tested for HIV  (N=9339) | HIV positive  (N=935) | HIV negative  (N=8404) | p-value |
| --- | --- | --- | --- | --- |
| Socio-demographics | | | | |
| Sex, n (%) |  |  |  |  |
| Male | 3886 (41.61) | 331 (35.40) | 3555 (42.30) | <0.001* |
| Female | 5453 (58.39) | 604 (64.60) | 4849 (57.70) |  |
| Median age (IQR) |  |  |  |  |
|  | 29 (23) | 41 (17) | 27 (22) | <0.001* |
| Age strata, n(%) |  |  |  |  |
| 15-19 | 2149 (23.01) | 51 (5.45) | 2098 (24.96) | <0.001* |
| 20-24 | 1671 (17.89) | 46 (4.92) | 1625 (19.34) | <0.001* |
| 25-29 | 788 (10.58) | 75 (8.02) | 913 (10.86) | 0.007* |
| 30-34 | 895 (9.58) | 116 (12.41) | 779 (9.27) | 0.002* |
| 35-39 | 803 (8.60) | 139 (14.87) | 664 (7.90) | <0.001* |
| 40-44 | 678 (7.26) | 146 (15.61) | 532 (6.33) | <0.001* |
| 45-49 | 566 (6.06) | 149 (15.94) | 417 (4.96) | <0.001* |
| 50-54 | 392 (4.20) | 104 (11.12) | 288 (3.43) | <0.001* |
| 55-59 | 287 (3.07) | 41 (4.39) | 246 (2.93) | 0.014* |
| 60-64 | 217 (2.32) | 30 (3.21) | 187 (2.23) | 0.058 |
| 65-69 | 274 (2.93) | 20 (2.14) | 254 (3.02) | 0.129 |
| 70+ | 419 (4.49) | 18 (1.93) | 401 (4.77) | <0.001* |
| Highest education level^δ^, n/N(%) |  |  |  |  |
| None/Primary | 1932/9180 (21.05) | 243/906 (26.82) | 1689/8274 (20.41) | <0.001* |
| Secondary/Higher | 7248/9180 (78.95) | 663/906 (73.18) | 8585/8274 (79.59) |  |
| Study site, n/N(%) |  |  |  |  |
| Eastern Highlands (rural) | 1006/9339 (10.77) | 117/935 (12.51) | 889/8404 (10.58) | 0.070 |
| Bonda (rural) | 1474/9339 (15.78) | 128/935 (13.69) | 1346/8404 (16.02) | 0.064 |
| Selbourne (rural) | 1356/9339 (14.52) | 145/935 (15.51) | 1211/8404 (14.41) | 0.366 |
| Nyazura (peri-urban) | 1397/9339 (14.96) | 185/935 (19.79) | 1212/8404 (14.42) | <0.001* |
| Nyanga (peri-urban) | 937/9339 (10.03) | 68/935 (7.27) | 869/8404 (10.34) | 0.003* |
| Watsomba (peri-urban) | 1656/9339 (17.73) | 154.935 (16.47) | 1502/8404 (17.87) | 0.287 |
| Sakubva (urban) | 743/9339 (7.96) | 82/935 (8.77) | 661/8404 (7.87) | 0.332 |
| Hobhouse (urban) | 770/9339 (8.24) | 56/935 (5.99) | 714/8404 (8.50) | 0.008* |
| Site type, n/N (%) |  |  |  |  |
| Small towns | 2334/9339 (24.99) | 253/935 (27.06) | 2081/8404 (24.76) | 0.124 |
| Agricultural estates | 2362/9339 (25.29) | 262/935 (28.02) | 2100/8404 (24.99) | 0.043* |
| Roadside settlements | 1656/9339 (17.73) | 154/935 (16.47) | 1502/8404 (17.87) | 0.287 |
| Subsistence farming areas | 1474/9339 (15.78) | 128/935 (13.69) | 1346/8404 (16.02) | 0.064 |
| Urban | 1513/9339 (16.20) | 138/935 (14.76) | 1375/8404 (16.36) | 0.207 |
| Employment sector, n/N(%) |  |  |  |  |
| Formal | 1604/9339 (17.18) | 199/935 (21.28) | 1405/8404 (16.72) | <0.001* |
| Informal sector (petty trading, subsistence farming) | 2003/9339 (21.45) | 242/935 (25.88) | 1761/8404 (20.95) | <0.001* |
| Student | 1620/9339 (17.35) | 35/935 (3.74) | 1585/8404 (18.86) | <0.001* |
| Unemployed | 4112/9339 (44.03) | 459/935 (49.09) | 3653/8404 (43.47) | 0.01* |
| Wealth status, n(%) |  |  |  |  |
| Poorest | 923/9336 (9.89) | 94/935 (10.05) | 829/8401 (9.87) | 0.854 |
| 2^nd^ poorest | 4219/9336 (45.19) | 464/935 (49.63) | 3755/8401 (44.70) | 0.004* |
| 3^rd^ poorest | 2102/9336 (22.51) | 210/935 (22.46) | 1892/8401 (22.52) | 0.971 |
| 4^th^ poorest | 1964/9336 (21.04) | 159/935 (17.01) | 1805/8401 (21.49) | 0.001* |
| Least poor | 128/9336 (1.37) | 8/935 (0.86) | 120.8401 (1.43) | 0.153 |
| Church denomination, n(%) |  |  |  |  |
| Christian | 4962/9339 (53.13) | 453/935 (48.45) | 4509/8404 (53.65) | 0.002* |
| Spiritualist | 2578/9339 (27.60) | 262/935 (28.02) | 2316/8404 (27.56) | 0.764 |
| Other | 1109/9339 (11.87) | 122/935 (13.05) | 987/8404 (11.74) | 0.242 |
| None | 690/9339 (7.39) | 98/935 (10.48) | 592/8404 (7.04) | <0.001* |
| Risk behaviours | | | | |
| Alcohol drinkers^ε^, n/N(%) | 1497/9337 (16.03) | 169.935 (18.07) | 1328/8402 (15.81) | 0.073 |
| Injecting drug users, n/N (%) | 9/9339 (0.10) | 0/935 (0.00) | 9/8404 (0.11) | N/A |
| Age of sexual debut, n/N (%) |  |  |  |  |
| <18 | 2017/9337 (21.60) | 272/935 (29.09) | 1745/8402 (20.77) | <0.001* |
| ≥18 | 5174/9337 (55.41) | 614/935 (65.67) | 4560/8402 (54.27) | <0.001* |
| Never had sex | 2146/9337 (22.98) | 49/935 (5.24) | 2097/8402 (24.96) | <0.001* |
| Number of sexual partners during lifetime, n/N (%)^φ^ |  |  |  |  |
| Total number (regular and non-regular), n/N (%) |  |  |  |  |
| 0 | 155/7190 (2.16) | 11/886 (1.24) | 144/6304 (2.28) | 0.046* |
| 1-4 | 6116/7190 (85.06) | 685/886 (77.31) | 5431/6304 (86.15) | <0.001* |
| 5+ | 919/7190 (12.78) | 190/886 (21.44) | 729/6304 (11.56) | <0.001* |
| Number of regular partners, n/N(%) |  |  |  |  |
| 0 | 438/7156 (6.12) | 25.879 (2.84) | 413/6277 (6.58) | <0.001* |
| 1-4 | 6554/7156 (91.59) | 817/879 (92.95) | 5737 (91.40) | 0.221 |
| 5+ | 164.7156 (2.29) | 37/879 (4.21) | 127/6277 (2.02) | <0.001* |
| Non-regular (any) | 2800/7193 (38.93) | 417/886 (47.07) | 2383/6307 (37.78) | <0.001* |
| Number of sexual partners during last year, n/N (%)^φ^ |  |  |  |  |
| Total number (regular and non-regular) |  |  |  |  |
| 1-4 | 7133/7188 (99.23) | 869/885 (98.19) | 6264/6303 (99.38) | <0.001* |
| 5+ | 55/7188 (0.77) | 16/885 (1.81) | 39/6303 (0.62) |  |
| Regular^ς^ |  |  |  |  |
| 0 | 479/5054 (9.48) | 65/576 (11.28) | 414/4478 (9.25) | 0.119 |
| 1-4 | 4568/5054 (90.38) | 510/576 (88.54) | 4058/4478 (90.62) | 0.225 |
| 5+ | 7/5054 (0.14) | 1/576 (0.17) | 6/4478 (0.13) | 0.810 |
| Non-regular^ς^ | 806/5539 (14.55) | 119/632 (18.83) | 687/4907 (14.00) | 0.001* |
| Concurrent sexual partnerships^φ^, n/N (%) | 220/7190 (3.06) | 38/886 (4.29) | 182/6304 (2.89) | 0.023* |
| Transactional sex^φγ^, n/N (%) |  |  |  |  |
| Males | 458/2468 (18.56) | 83/287 (28.92) | 375/2181 (17.19) | <0.001* |
| Females | 365/4034 (9.05) | 95/562 (16.90) | 270/3472 (7.78) | <0.001* |
| Recent STI symptoms^ψ^, n/N (%) | 433/7193 (6.02) | 89/886 (10.05) | 344/6307 (5.45) | <0.001* |
| Prevention methods | | | | |
| Condom use^φ^, n/N (%) |  |  |  |  |
| Throughout most recent sexual encounter | 1381/7190 (19.21) | 397/886 (44.81) | 984/6304 (15.60) | <0.001* |
| With last non-regular partner^σ^ | 948/2109 (44.95) | 119/328 (36.28 | 829/1781 (46.55) | 0.001* |
| Circumcision, n/N (%) |  |  |  |  |
| Medical | 816/3885 (21.00) | 10/331 (3.02) | 806/3554 (22.68) | <0.001* |
| Traditional/religious | 113/3885 (2.91) | 20/331 (6.04) | 93/3554 (2.62) | <0.001* |
| Both | 1/3885 (0.03) | 0/331 (0.00) | 1/3554 (0.03) | N/A |
| PrEP (ever), n/N (%) | 26/724 (3.59) | 1/104 (0.96) | 25/620 (4.03) | N/A |

**S1 Table. Socio-demographic characteristics and risk behaviours of study population by HIV status.** PrEP= Pre-exposure prophylaxis. P-values represent results of Chi-squared (categorical) or Mann-Whitney U tests (continuous, non-parametric). Asterisks (*) represents p<0.05. Where denominators are less than N, missing data is truly missing (omitted due to low numbers) and/or arising from skip rules in the questionnaire (explained below):

δ Restricted to participants currently not enrolled in school, including those aged 65+

ε Alcohol drinkers included participants who reported having any drink in the past year or having been to a bar/beer hall in the past month

^φ^ Questions on sexual partners were restricted to those who reported ever having sex

**^ς^** Restricted to participants reporting at least one sexual partner in the last year

γ Transactional sex was defined as having ever been involved in a non-marital relationship where participants gave something in exchange for sex or having given/received money in exchange for sex in participants’ past 3 sexual relationships. This was restricted to participants aged <65 years.

^ψ^ Self-reported STI symptoms in the last 12 months including discharge, pain and genital sores; restricted to sexually active participants.

σ Results shown for those participants who reported having at least one non-regular sexual partner in their lifetime or of their past 3 sexual partners.
